# Personality traits mediate the association between perceived parental bonding and well-being in adult volunteers from the community

**DOI:** 10.1186/s13030-020-00198-4

**Published:** 2020-10-19

**Authors:** Akiko Murakoshi, Nobuyuki Mitsui, Jiro Masuya, Yota Fujimura, Shinji Higashi, Ichiro Kusumi, Takeshi Inoue

**Affiliations:** 1 Department of Psychiatry, Tokyo Medical University, 6-7-1 Nishishinjuku, Shinjuku-ku, Tokyo, 160-0023 Japan; 2 Department of Psychiatry, Hokkaido University Graduate School of Medicine, North 15, West 7, Kita-ku, Sapporo, 060-8638 Japan; 3 Department of Psychiatry, Tokyo Medical University Hachioji Medical Center, Tokyo, 193-0998 Japan; 4 Department of Psychiatry, Ibaraki Medical Center, Tokyo Medical University, Ami-machi, Inashiki-gun, Ibaraki, 300-0395 Japan

**Keywords:** Subjective well-being, Quality of parenting, Temperament and character inventory, Structural equation model, Covariance structure analysis

## Abstract

**Background:**

Previous studies reported that subjective well-being in adulthood correlates with perceived parental bonding in childhood as well as personality traits. However, whether personality traits mediate the effect of perceived parental bonding on well-being or not has not been reported to date. In this study, we hypothesized that ‘parental care and overprotection’ in childhood affect ‘well-being’ in adulthood through various ‘personality traits’, and analyzed this using structural equation modeling.

**Methods:**

A total of 402 adult volunteers from the community provided responses to the following questionnaires: 1) Parental Bonding Instrument, 2) Temperament and Character Inventory, and 3) The Subjective Well-being Inventory. Two structural equation models were designed and the maximum likelihood estimation method was used for covariance structure analysis.

**Results:**

Parental care in childhood directly increased well-being in adulthood and indirectly increased it through personality traits (harm avoidance, reward dependence, and self-directedness). Parental overprotection in childhood had no direct effect on well-being in adulthood but decreased well-being in adulthood indirectly through personality traits (harm avoidance, reward dependence, and self-directedness) and increased it through one personality trait (self-transcendence).

**Conclusions:**

This study showed that the influences of perceived parental bonding on well-being in adulthood are mediated by self-directedness, harm avoidance, reward dependence, and self-transcendence among the seven personality dimensions evaluated by the Temperament and Character Inventory.

## Background

In psychiatric treatments, such as pharmacotherapy and psychotherapy, ill-being, such as depressive and anxious symptoms and fatigability, are treated thoroughly, whereas the promotion of well-being has been ignored. The constitution of the World Health Organization defined health as a state of complete physical, mental, and social well-being, not merely the absence of disease or infirmity, and indicated the importance of the promotion of well-being as well as the treatment of ill-being [1, 2]. External factors (environment and stress) and internal factors (personality traits) that influence well-being have been investigated, but there is presently an insufficient amount of data [3–7].

Various personality traits have important influences not only on ill-being, such as depression and anxiety [8, 9], but also on well-being [6, 10–14]. The previous studies investigated the influence of seven personality traits evaluated by the Temperament and Character Inventory (TCI) on well-being [15] and found reproducible results that high self-directedness and high cooperativeness are associated with high well-being and that high harm avoidance is associated with low well-being [10–14]. On the other hand, several studies analyzed the association between five personality traits measured by the ‘Big Five’ personality model and well-being. Meta-analysis studies demonstrated that neuroticism was negatively correlated with well-being and positively correlated with the other four personality traits, but the weighted and unweighted estimates for each personality variable correlated with well-being were different [16]. Furthermore, in the five affective temperament dimensions of Temperament Evaluation of Memphis, Pisa, Paris, and San Diego auto-questionnaire version (TEMPS-A), four affective temperaments (depressive, cyclothymic, irritable, and anxious) were negatively associated with well-being, but hyperthymic temperament was positively associated with well-being [6].

Previous studies reported that perceived parental bonding during childhood influences personality traits of the ‘Big Five’ model and the TCI [17, 18]. Early experiences of parental bonding and abuse from parents during childhood are expected to affect well-being in adulthood, but only a few studies on this point have been reported to date [6, 19, 20]. Furthermore, because there is a long time interval between early childhood experiences and well-being in adulthood, some factors may mediate the association between early childhood experiences and adulthood well-being [6, 21]. A long-term prospective study by Huppert and colleagues reported that perceived parental bonding, which consists of two subscales of care and overprotection, scored by the Parental Bonding Instrument (PBI) [22], influenced well-being in adulthood, and this effect was mediated by neuroticism and extraversion scored by the Maudsley Personality Inventory (MPI) [21]. This study demonstrated that higher levels of parental care were associated with higher psychological well-being, whereas higher parental non-engagement or control was associated with lower levels of psychological well-being [21]. Furthermore, we reported that cyclothymic, anxious, and irritable temperaments measured by the TEMPS-A were mediators in the influence of childhood abuse on adulthood well-being [6]. However, in our previous study, childhood abuse was not limited to abuse by parents [6]. In general, human personality can be explained by five or more dimensions, such as the ‘Big Five’ personality model and the seven dimensions of the TCI [16, 23]. However, the mediating effects of personality traits between parenting and well-being have only been reported for a few personality traits, including neuroticism and extraversion [6, 21], and have not been studied regarding a larger number of personality traits using comprehensive personality models, such as the TCI and the ‘Big Five’. Therefore, the mediating effects of other basic personality traits between parenting and well-being should be analyzed using comprehensive personality models.

In this study, we hypothesized that ‘parental care and overprotection’ in childhood affect ‘well-being’ in adulthood through various ‘personality traits’ of the TCI, which is a comprehensive personality model. We chose to use the TCI in this study for the following reasons. First, a close association between subjective well-being, temperament, and character dimensions has been consistently found using the TCI [10–14]. Second, to our knowledge the mediating effects of personality dimensions of the TCI on the effects of perceived parenting on subjective well-being have not yet been reported. Third, dimensions of temperament and character may be differently associated with perceived parental bonding and subjective well-being; i.e., temperament is an innate quality or tendency, whereas character is created by a person’s own volition [15]. Fourth, although character dimensions, which are important aspects of personality development that are influenced by experiences in early childhood should be evaluated, they cannot be evaluated by the Big Five model [24]. To verify this hypothesis, ‘parental care and overprotection’ were evaluated by the PBI [22], ‘personality traits’ were evaluated by the TCI [15], and ‘well-being’ (positive affect) was evaluated by the Subjective Well-being Inventory (SUBI) [1]. Furthermore, correlations between the variables and multiple regression analysis using well-being as a dependent variable were performed, and the interrelationships between variables, especially mediation, were analyzed using structural equation modeling (SEM).

## Methods

### Participants

Between January and August 2014, questionnaires were distributed to 853 Japanese volunteers who were recruited by convenience sampling through our colleagues at Hokkaido University. This study was part of a larger study [6]. Of the 853 volunteers, 415 agreed to participate in this study and provided written informed consent, 402 of whom (97%) provided complete responses to the three questionnaires on parenting, personality, well-being, and demographic characteristics (age, sex, etc.). The completed questionnaires were returned by mail to maintain complete confidentiality. The ethics review boards of Hokkaido University Hospital (study approval no. 013–0184) and Tokyo Medical University (study approval no. SH3308) approved this study.

### Questionnaires

#### Parental Bonding Instrument (PBI)

The PBI retrospectively evaluates perceived parental bonding to the child [22]. Subjects answer 25 questions regarding the care (12 items) and overprotection (13 items) they experienced until the age of 16 years. In this study the Japanese version of the PBI was used, which was developed by Kitamura and Suzuki and has been confirmed regarding its validity and reliability [25].

#### Temperament and Character Inventory (TCI)

Cloninger and colleagues hypothesized the structural concept of personality, proposed a seven-dimensional model, which was constructed of four-temperament dimensions and three-character dimensions, and developed the self-rating questionnaire of the TCI [15]. There are four dimensions of temperament: novelty seeking, reward dependence, harm avoidance, and persistence. There are three dimensions of character: self-directedness, cooperativeness, and self-transcendence. The Japanese version of the TCI was developed by Kijima et al. [26] and its validity and reliability were confirmed [27]. The present study administered the 125-item TCI with a four-point scale. Kijima et al. showed that a four-point scale was superior to a dichotomous scale in terms of internal consistency, as expressed by Cronbach’s α coefficients [26, 27].

#### Subjective well-being inventory (SUBI)

The SUBI, which was developed by the World Health Organization [1], is a 40-item self-reported questionnaire consisting of items regarding subjective well-being (19 items) and subjective ill-being (21 items). The Japanese version of the SUBI used in this study was developed by Ono and colleagues, and its validity and reliability were confirmed [28, 29]. In this study, only the well-being subscale was used for the statistical analysis.

### Data analysis

The effects of demographic variables (sex, marital status, employment status, history of psychiatric illness, and a first-degree relative with psychiatric illness) on well-being in the SUBI were analyzed by the Student *t*-test. Demographic characteristics (age, education years, number of cohabiters, and number of offspring), questionnaire data (PBI and TCI), and well-being in the SUBI were analyzed using Pearson correlation coefficients.

The results of univariate analyses may be affected by confounding factors. Therefore, to eliminate their effects and to analyze the effects of multiple factors, multiple regression analysis was conducted. Stepwise multiple regression analysis with backward elimination was performed with demographic data and questionnaire (PBI and TCI) data as independent variables and the score of subjective well-being on SUBI as a dependent variable, using SPSS 22.0 J software.

After the confirmation and selection of factors associated with well-being, the mediation between parenting and well-being by seven personality dimensions was analyzed by structural equation modeling using latent variables consisting of factors of the mother and father. In the two structural equation models, subjective well-being of the SUBI was predicted by perceived parental bonding (PBI, care or overprotection) and personality traits (TCI). Parental care and parental overprotection consist of observed paternal and maternal variables. Because overprotection and care sometimes show different effects [30] and high/low overprotection scores and high/low care scores compose distinctive quadrants in the comparison of controls vs. depressive patients, indicating an orthogonal association [31], the mediating effects of personality traits (TCI) were analyzed separately for overprotection and care. Furthermore, because this study intended to analyze the total effects of paternal and maternal parenting on personality traits and well-being, paternal and maternal variables were combined as a latent variable rather than being separated.

**Fig. 1:**
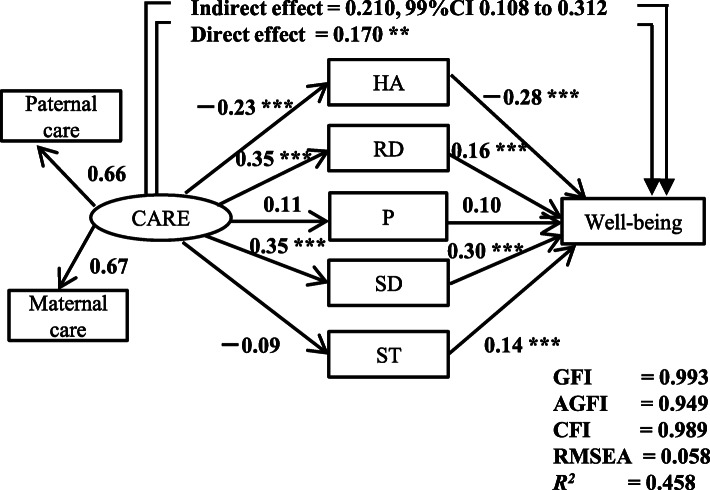
The structural equation model including ‘care’ of the PBI, personality trait subscales of the Temperament and Character Inventory, i.e., harm avoidance (HA), reward dependence (RD), persistence (P), self-directedness (SD), and self-transcendence (ST), as well as the well-being subscale of the Subjective Well-being Inventory from 402 adult volunteers. Rectangles indicate the observed variables, and the oval indicates the latent variable. The numbers indicate the direct standardized path coefficients. The indirect effect of ‘care’ on ‘well-being’ through five personality traits indicates the standardized coefficient with a 99%CI calculated by Bayesian estimation. **p < 0.01, ***p < 0.001. GFI, Goodness of Fit Index; AGFI, Adjusted GFI; CFI, Comparative Fit Index; RMSEA, Root Mean Square Error of Approximation

Model 1 (Fig. 1): Parental care increases subjective well-being directly and also affects subjective well-being indirectly through its influence on personality traits.

**Fig. 2:**
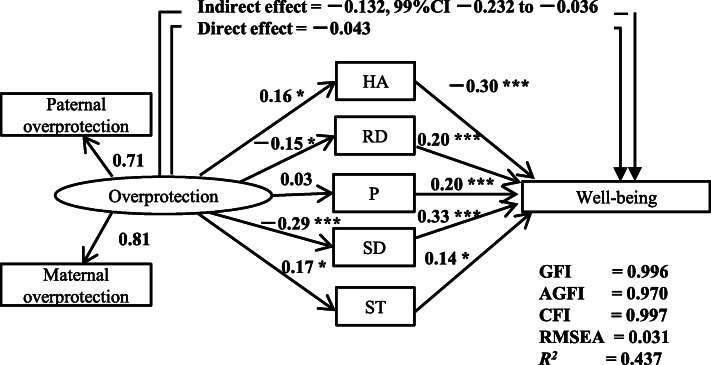
The structural equation model including ‘overprotection’ of the PBI, personality trait subscales of the Temperament and Character Inventory, i.e., harm avoidance (HA), reward dependence (RD), persistence (P), self-directedness (SD), and self-transcendence (ST), as well as the well-being subscale of the Subjective Well-being Inventory from 402 adult volunteers. Rectangles indicate the observed variables, and the oval indicates the latent variable. The numbers indicate the direct standardized path coefficients. The indirect effect of ‘overprotection’ on ‘well-being’ through five personality traits indicates the standardized coefficient with the 99%CI calculated by Bayesian estimation. *p < 0.05, ***p < 0.001

Model 2 (Fig. 2): Parental overprotection decreases subjective well-being directly and also affects subjective well-being indirectly through its influence on personality traits.

AMOS 22.0 J software (SPSS, Chicago, IL, USA) was used to analyze structural equation models with the maximum likelihood estimation method. The direct and indirect effects of all the variables were calculated. The Goodness of Fit Index (GFI), the Adjusted GFI (AGFI), the Comparative Fit Index (CFI), and the Root Mean Square Error of Approximation (RMSEA) were used as indices of goodness of fit. A GFI > 0.90, an AGFI > 0.85, a CFI > 0.95, and an RMSEA < 0.08 indicate an acceptable fit; a GFI > 0.95, an AGFI > 0.90, a CFI > 0.97, and an RMSEA < 0.05 indicate a good fit [32].

Finally, we analyzed the indirect effect of perceived parental bonding (PBI) on well-being in the SUBI through each personality trait (TCI). Bayesian estimation calculated the 95 and 99% confidence intervals (CI) of the indirect effect using the Markov Chain Monte Carlo approach with more than 100,000 iterations after a burn-in phase of 1000 iterations and a Gelman-Rubin potential scale reduction factor < 1.002.

The threshold for significance was set at *p* less than 0.05.

## Results

### Demographic characteristics and PBI, TCI, and SUBI scores

The demographic characteristics, parental care and overprotection scores of the PBI, personality trait subscale scores of the TCI, and subjective well-being scores of the SUBI in 402 adult volunteers are presented in Table 1.

**Table 1:** Characteristics, PBI, TCI, and correlation with well-being scores of the SUBI or effects on well-being scores of the SUBI in 402 subjects from the general adult population

Characteristic or measure	Value (number or mean ± SD)	Correlation with well-being scores (*r*) or effect on well-being scores (mean score, *t*-test)
Age	42.1 ± 11.8	*r* = − 0.155^**^
Gender (male:female)	220:182	Male = 39.4 ± 6.6; Female = 38.8 ± 6.2, n.s. (*t*-test)
Education years	15.2 ± 2.0	*r* = 0.162^**^
Marital status (married:unmarried)	286:113	Married = 39.3 ± 6.3; Unmarried = 38.4 ± 6.4, n.s. (*t*-test)
Employment status (employed:non-employed)	339:56	Employed = 39.0 ± 6.3; Non-employed = 39.6 ± 7.4, n.s. (*t*-test)
Number of cohabiters	1.8 ± 1.5	*r* = 0.08, n.s.
Number of offspring	1.3 ± 1.2	*r* = 0.05, n.s.
History of psychiatric illness (yes:no)	18:384	Yes = 36.5 ± 7.8; No = 39.2 ± 6.3, n.s. (*t*-test)
First-degree relative with psychiatric illness (yes:no)	40:360	Yes = 37.7 ± 7.1; No = 39.3 ± 6.3, n.s. (*t*-test)
Well-being score on SUBI	39.1 ± 6.4	
PBI (subscale score)		
Paternal care	24.0 ± 7.2	*r* = 0.244^**^
Maternal care	27.7 ± 6.7	*r* = 0.278^**^
Paternal overprotection	9.4 ± 6.0	*r* = − 0.124^*^
Maternal overprotection	10.2 ± 6.8	*r* = − 0.155^**^
TCI (subscale score)		
Novelty seeking	28.3 ± 6.2	*r* = 0.111^*^
Harm avoidance	30.7 ± 8.1	*r* = − 0.514^**^
Reward dependence	26.9 ± 5.0	*r* = 0.343^**^
Persistence	7.5 ± 2.2	*r* = 0.259^**^
Self-directedness	45.8 ± 9.3	*r* = 0.515^**^
Cooperativeness	46.6 ± 7.4	*r* = 0.310^**^
Self-transcendence	11.8 ± 5.9	*r* = 0.093, n.s.

Data are presented as means ± SD or numbers

*r* = Pearson correlation coefficient

*SUBI* Subjective Well-being Inventory, *PBI* Parental Bonding Instrument, *TCI* Temperament and Character Inventory

* *P* < 0.05

** *P* < 0.01

*n.s.* not significant

In the correlation analysis, age was negatively correlated with well-being and education years were positively correlated with well-being. The other demographic characteristics were not significantly correlated with well-being. Paternal and maternal care scores were positively correlated with well-being scores, but paternal and maternal overprotection scores were negatively correlated with well-being scores. A harm avoidance score of the TCI was negatively correlated with well-being; however, on the contrary, novelty seeking, reward dependence, persistence, self-directedness, and cooperativeness scores were positively correlated with well-being scores. The self-transcendence score was not correlated with the well-being score. In particular, the correlation of harm avoidance and self-directedness scores with well-being scores were very high (*r* = − 0.514 and 0.515, respectively).

### Stepwise multiple regression analysis of SUBI well-being scores

In stepwise multiple regression analysis, a subjective well-being score of the SUBI was the dependent variable, and several demographic variables, such as age, sex, etc., PBI scores (care and overprotection by father and mother), and TCI scores (novelty seeking, harm avoidance, reward dependence, persistence, self-directedness, cooperativeness, and self-transcendence) were independent variables (Table 2). Marital status (married), maternal care, reward dependence, persistence, self-directedness, and self-transcendence were positively associated with the SUBI well-being score, but age and harm avoidance were negatively associated with the SUBI well-being score (adjusted *R*^2^ = 0.487, F = 45.414, *p* <  0.001). Theses variables predicted the SUBI well-being score independently. Independent variables, which strongly affect well-being, were age, harm avoidance, and self-directedness. Multicollinearity was denied.

**Table 2:** The results of a stepwise multiple regression analysis of the SUBI well-being score

Characteristic	Beta	*P*-value	VIF
Age	−0.306	< 0.001	1.169
Married	0.098	0.012	1.092
PBI Maternal care score	0.118	0.003	1.109
TCI Harm avoidance	−0.306	< 0.001	1.534
Reward dependence	0.159	< 0.01	1.214
Persistence	0.103	0.010	1.159
Self-directedness	0.332	< 0.001	1.871
Self-transcendence	0.141	0.001	1.287

*Beta* standardized partial regression coefficient, *VIF* Variance Inflation Factor, *SUBI* Subjective Well-being Inventory, *PBI* Parental Bonding Instrument, *TCI* Temperament and Character Inventory

Dependent variable: SUBI well-being score

Nineteen independent variables: age, sex (male = 0, female = 1), marital status (unmarried = 0, married = 1),

number of offspring, living alone (yes = 0, no = 1), education years, employment status (unemployed = 0, employed = 1), past history of psychiatric illness (yes = 0, no = 1), first-degree relative with psychiatric illness (yes = 0, no = 1), PBI scores (paternal care, maternal care, paternal overprotection, maternal overprotection), TCI scores (novelty seeking, harm avoidance, reward dependence, persistence, self-directedness, cooperativeness, self-transcendence)

Eight variables in Table 2 were significant variables by the results of stepwise multiple regression analysis

Adjusted *R*^2^ = 0.487, F = 45.414

*P* < 0.001

### Analysis of structural equation model 1

An acceptable fit was obtained for model 1; GFI = 0.993, AGFI = 0.949, CFI = 0.989, and RMSEA = 0.058 (Fig. 1). Paternal care from the latent variable ‘care’ was similar to maternal care in the standardized coefficients. In this model, direct positive effects of care on the SUBI well-being score were statistically significant (standardized path coefficient: 0.170, *p* <  0.01). Furthermore, an indirect effect of care on the SUBI through all personality traits by Bayesian estimation was statistically significant (0.210, 99%CI: 0.108–0.312). Care had a significant negative effect on harm avoidance (− 0.23, *p* <  0.001) and significant positive effects on reward dependence and self-directedness (0.35 and 0.35, respectively, *p* <  0.001). Regarding the effects of personality traits on the SUBI well-being score, harm avoidance had a significant negative effect on well-being (− 0.28, *p* <  0.001), and reward dependence, self-directedness, and self-transcendence had a significant positive effect on well-being (0.16, 0.30, and 0.14, respectively, *p* <  0.001). The square of the multiple correlation coefficient of the SUBI well-being score in this model was 0.458. In other words, 45.8% of the variability of the well-being score was explained by this model.

The results of the indirect effect of care (PBI) on well-being (SUBI) through each personality trait (TCI) by Bayesian estimation are shown in Table 3. Parental care had indirect positive effects on well-being through personality traits of harm avoidance, reward dependence, and self-directedness (*p* <  0.01).

**Table 3:** The 95 and 99% confidence intervals of the standardized path coefficients of the indirect effect of care and overprotection of the PBI on well-being of the SUBI through subscales of the TCI

Care of the PBI				Overprotection of the PBI		
Coefficients		Lower limit	Upper limit	Coefficients	Lower limit	Upper limit
95% confidence interval						
HA	0.094	0.032	0.155	−0.067	− 0.127	− 0.012
RD	0.080	0.043	0.123	−0.043	−0.084	− 0.009
P	0.021	−0.007	0.051	0.015	−0.010	0.041
SD	0.147	0.094	0.203	−0.137	−0.200	− 0.077
ST	−0.012	− 0.036	0.003	0.021	0.002	0.049
99% confidence interval						
HA	0.097	0.022	0.169	−0.070	−0.149	−0.002
RD	0.080	0.031	0.141	−0.042	−0.097	0.002
P	0.020	−0.018	0.062	0.014	−0.020	0.051
SD	0.149	0.080	0.228	−0.137	−0.222	− 0.063
ST	−0.013	− 0.054	0.008	0.021	−0.002	0.061

Lower limit and upper limit indicate 95% confidence interval of the indirect effect

*PBI* Parental Bonding Instrument, *SUBI* Subjective Well-Being Inventory, *TCI* Temperament and Character Inventory, *HA* Harm avoidance, *RD* Reward dependence, *P* Persistence, *SD* Self-directedness, *ST* Self-transcendence

In summary, parental care in childhood directly increased well-being in adulthood and indirectly increased adulthood well-being through personality traits (harm avoidance, reward dependence, and self-directedness).

### Analysis of structural equation model 2

A good fit was obtained for model 2; GFI = 0.996, AGFI = 0.970, CFI = 0.997, and RMSEA = 0.031 (Fig. 2). Paternal overprotection from the latent variable ‘overprotection’ was similar to maternal overprotection in standardized coefficients. In this model, a direct effect of overprotection on the SUBI well-being score was not statistically significant. On the other hand, indirect effects of overprotection on the SUBI through all personality traits by Bayesian estimation were statistically significant (− 0.132, 99%CI: − 0.232 to − 0.036). Overprotection had significant positive effects on harm avoidance and self-transcendence (0.16 and 0.17, respectively, *p* <  0.05) and significant negative effects on reward dependence and self-directedness (− 0.15, *P* < 0.05 and − 0.29, *p* < 0.001, respectively). Regarding the effects of personality traits on the SUBI well-being score, harm avoidance had a significant negative effect on well-being scores (− 0.30, *p* < 0.001) and reward dependence, persistence, and self-directedness (0.20, 0.20, and 0.33, *p* < 0.001, respectively) and self-transcendence (0.14, *p* < 0.05) had significant positive effects on well-being scores. The square of the multiple correlation coefficient of the SUBI well-being score in this model was 0.437. In other words, 43.7% of the variability of well-being scores was explained by this model.

The results of the indirect effects of overprotection (PBI) on well-being of SUBI through each personality trait (TCI) by Bayesian estimation are shown in Table 3. Parental overprotection had indirect negative effects on well-being scores through each personality trait of harm avoidance, reward dependence, and self-directedness (*p* < 0.01, *p* < 0.05, and *p* < 0.01, respectively), but had an indirect positive effect on well-being scores through a personality trait of self-transcendence (*p* < 0.05).

In summary, childhood parental overprotection had no direct effect on adulthood well-being. However, childhood parental overprotection decreased adulthood well-being indirectly through personality traits (harm avoidance, reward dependence, and self-directedness) and increased adulthood well-being indirectly through one personality trait (self-transcendence).

## Discussion

To our knowledge, this study is the first report to date showing that perceived parental bonding (care and overprotection as evaluated by the PBI) in childhood indirectly affects adulthood well-being (as evaluated by the SUBI) through personality traits (as evaluated by the TCI), using covariance structure analysis for 402 adult volunteers from a community in Japan. Although an earlier study showed the mediating effects of a limited number of personality traits, including neuroticism and extraversion [6, 21], in the present study we analyzed the mediating effects of a large number of personality traits using a comprehensive personality model, which is the strength of this study.

The association between personality traits and well-being was reported in some previous studies [10–14]. Self-directedness was strongly associated with various aspects of well-being, whereas harm avoidance was negatively associated with well-being [11, 14]. A few studies have noted a correlation between perceived parental bonding and personality traits. Lower parental care was associated with higher harm avoidance and lower self-directedness; higher parental overprotection was associated with higher harm avoidance [17, 18]. High parental care was associated with high psychological well-being, whereas high parental non-engagement and control were associated with low levels of psychological well-being [21]. The associations between personality and well-being, between parenting and personality, and between parenting and well-being were confirmed in our present study, indicating the reliability of our findings as well as those of the previous study.

To our knowledge, there has been no study to date investigating the indirect effects of perceived parental bonding on well-being and its mediation by personality traits as evaluated by the TCI, which is a comprehensive personality model. Only a few studies reported that the experiences of childhood (parenting and abuse) indirectly affected well-being in adulthood through personality traits, as evaluated by the MPI and the TEMPS-A [6, 21]. As mentioned above, one study reported that the effects of parental care and higher non-engagement or control on psychological well-being were largely mediated by the personality traits of neuroticism and extraversion, as scored by the MPI [21]. Another of our previous study demonstrated that child abuse worsened the well-being of individuals indirectly through affective temperament as scored by TEMPS-A [6]. Our present study is the first report to show, using a structural equation model, that perceived parental bonding in childhood affects well-being in adulthood through personality traits as evaluated by the TCI. The mediating effects of personality traits on adulthood well-being were different for childhood parental care and overprotection; these were both mediated by harm avoidance, reward dependence, and self-directedness, whereas self-transcendence only mediated the effects of overprotection. However, other personality traits in a seven-dimensional model of personality (four dimensions of temperament and three dimensions of character) [15] did not show any mediating effects. Therefore, various personality dimensions play different roles in the association between parenting and well-being.

Cloninger stated that well-being and happiness are not achieved by conventional psychiatric or psychological treatments and that psychiatry should focus on the understanding and development of positive mental health [3, 4]. Indeed, psychiatrists generally do not consider well-being as a treatment target [33]. Based on their research, Cloninger proposed the psychotherapy program ‘coherence therapy’, which includes psychoeducation to promote self-awareness and to improve well-being [3, 4, 8]. Because we found in this study that personality traits on the TCI link perceived parental bonding to well-being, we should consider the associations among personality, parental bonding, and well-being when treating patients with the goal of achieving well-being.

There are some limitations to this study. Firstly, perceived parental bonding was retrospectively evaluated, which may hence be affected by recall bias. Nevertheless, previous studies showed that PBI scores remain unchanged over the years and correlate with objective evaluations [22, 25, 34]. Secondly, because this study was cross-sectional, causal associations cannot be concluded. Thirdly, the subjects were adult volunteers from the community of a limited area. Therefore, to confirm the results of this study, a long-term prospective study on a large number of subjects from many areas of Japan should be performed in the future.

## Conclusion

This study showed that the influences of perceived parental bonding on well-being in adulthood are mediated by self-directedness, harm avoidance, reward dependence, and self-transcendence among the seven personality dimensions evaluated by the TCI. In the future, the promotion of well-being as well as the treatment of ill-being is necessary in psychiatric care.

## Data Availability

Detailed data are available from the corresponding authors upon reasonable request.

## Abbreviations

MPI: Maudsley Personality Inventory
PBI: Parental Bonding Instrument
SEM: Structural Equation Modeling
SUBI: Subjective Well-being Inventory
TCI: Temperament and Character Inventory
TEMPS-A: Temperament Evaluation of Memphis, Pisa, Paris, and San Diego auto-questionnaire version
